# Intra-articular injection of loaded sPL sustained-release microspheres inhibits osteoarthritis and promotes cartilaginous repairs

**DOI:** 10.1186/s13018-021-02777-9

**Published:** 2021-10-30

**Authors:** Jiyou Li, Ning Liu, Zhipeng Huang, Wantao Wang, Donghua Hou, Wenbo Wang

**Affiliations:** The First Affiliated Hospital of Harbin Medical University, 23 You Zheng Street, Harbin, 150001 China

**Keywords:** Platelet-rich lysate, Osteoarthritis, PLGA

## Abstract

**Background:**

Osteoarthritis is a chronic inflammatory disease of the joints associated with significant morbidity and lower quality of life. Current treatment strategies focus on reducing cartilage degeneration but fail to restore their proliferative ability. Super-activated platelet lysate (sPL) is an enhanced form of platelet-rich plasma that can be easily inactivated. The purpose of this study is to evaluate whether sPL-loaded PLGA/chitosan/gelatin microspheres can prevent and treat osteoarthritis.

**Methods:**

Features of biological microspheres were detected by SEM and ELISA. Osteoarthritis chondrocytes were co-cultured with hydrogel loaded with sPL. The effect of biological microspheres on chondrocyte proliferation was evaluated using a CCK-8 cell proliferation test. Cell morphology and cell necrosis were measured with a microscope. The gene expression levels of cartilage-related markers type 2 collagen, aggrecan (ACAN), and SRY type high mobility group box-9 (SOX9) were determined by real-time quantitative polymerase chain reaction (Rt-PCR). A rat osteoarthritis model was established. Micro-CT was used to characterize cartilaginous changes after the injection of biological microspheres. Histopathological HE staining, Safranin-O Fast Green staining and staining scores, type II collagen staining, and proteoglycan staining were used to evaluate the degree of cartilaginous repair.

**Results:**

Biological microspheres were able to continuously release biological factors. Exposure to loading sPL microspheres significantly increased chondrocyte proliferation, reduced cell necrosis, and increased the expression of cartilage markers type 2 collagen, ACAN, and SOX9 in osteoarthritic chondrocytes. In vivo experiments found that biological microspheres also smoothen cartilage surfaces, promote the expression of proteoglycan and type 2 collagen while also increasing cartilaginous integrity as evaluated using Safranin-O Fast Green staining.

**Conclusions:**

PLGA/chitosan/gelatin hydrogel loaded with sPL is a promising tool for effective and non-invasive articular cartilage repair in osteoarthritis.

**Graphic abstract:**

Biological microspheres loaded with sPL release various biological factors to promote chondrocyte proliferation and upregulate chondrocyte functionalization genes (SOX9, CoX II, ACAN), leading to an overall enhanced cartilaginous matrix.
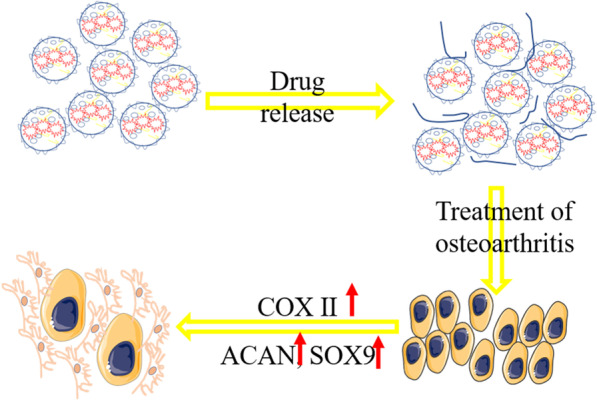

## Introduction

Osteoarthritis (OA) is a chronic inflammatory condition of the joints marked by cartilage degeneration. Cartilage is an avascular tissue composed of chondrocytes, which account for 2–3% of the total cartilage volume, and produce the extracellular matrix (ECM) that is rich in collagen and proteoglycans. Osteoarthritic cartilage repair may be facilitated by the use of tissue engineering, which incorporates cells, growth factors, and biomaterials to create or mimic cartilage tissue. PLGA is routinely used in biomedical applications as a carrier for delivering drugs and other small molecular weight compounds, such as peptides and proteins [1, 2]. Several PLGA-based preparations have been approved by the US Food and Drug Administration (FDA) for clinical use [3, 4]. Chitosan (CS) is a natural polysaccharide composed of glucosamine and N-acetylglucosamine. It is structurally similar to glycosaminoglycan (GAG) and represents a suitable carrier for drug delivery, gene therapy and tissue engineering [5–7]. Water-soluble CS is a more biocompatible version of CS and is retained for a longer duration in tissues [8–10].

Gelatin is a natural protein derived from partially denatured collagen and is a highly promising scaffold material for biomedical applications given its excellent biodegradable, biocompatible, cell adhesive, non-toxic, and non-immunogenic properties. However, its clinical use is limited due to poor mechanical properties, inconsistent solubility, and rapid degradation [11].

Hydrophilic polymers offer several advantages as drug carriers, such as enhanced water solubility, pharmacokinetics, and biodistribution of carried drugs, along with consistent drug delivery. Li et al. observed prolonged and controlled release of bovine serum albumin (BSA)-loaded dextran particles when embedded in a nanofiber PGLA scaffold [12]. Furthermore, growth factors coated with polymer microspheres can effectively promote cell adhesion, proliferation, and growth during tissue engineering. Xingquan et al. recently demonstrated that non-protein small molecule cattorkinin (KGN) induced chondrogenesis in damaged cartilage and promoted tissue repair by increasing TGFβ1 secretion and spatial distribution [13].

Super-active platelet lysate (sPL) contains high concentrations of over 150 bioactive factors, including TGFβ1, TGFβ3, insulin-like growth factor (IGF-1), and VEGF, all of which are critical in cartilage repair. Growth factors released from the platelets adhere immediately to cell surfaces via specific receptors, stimulate cell signal transduction, and activate transcription of genes related to chondrocyte proliferation, osteoid production, and matrix formation.

We encapsulated sPL containing various biologically active factors into microspheres composed of water-soluble CS, gelatin and PLGA. These microspheres can not only mimic the extracellular matrix but also continuously release sPL biological factors to repair osteoarthritis cartilage defects by intra-articular injection. Therefore, this study proves that hydrophilic microspheres loaded with sPL are a promising option for regenerating damaged cartilage in OA.

## Materials and methods

### sPL production and synthesis

sPL was prepared using a patented technology as previously reported [14]. Briefly, whole blood was centrifuged at 2000*g* for 10min. Platelet-rich plasma (PRP) was obtained by lysing platelets with repeated freeze-thaw cycles. Biological factor culture technology was then employed to induce and activate sPL.

### Synthesis of sPL/CS/gelatin nanospheres

1 g PLGA (Daigang BIO Engineer Limited Co. Ltd. China) was dissolved in 11 ml dichloromethane (Aladdin, China) and constantly stirred for 4 h. After dissolving 2 g water-soluble CS (BOSF) in 11 ml deionized water for 1 h, 0.1 g gelatin (Beigang Haituo Experimental Instrument in Zhonglou District, China) was added. The mixture was stirred for 1 h. Subsequently, 3 ml of CS and gelatin mixture was slowly added to 3 ml PLGA, followed by 500 µl or 1000 µl sPL. The respective mixtures were centrifuged at 600 rpm for 2 min, and the L0 (no sPL), L1 (500 µl sPL), and L2 (1000 µl sPL) nanosphere precipitates were isolated. Prior to the cellular assays, the nanospheres were sterilized by ultraviolet irradiation, immersed in ethanol (75%), and thoroughly washed with sterile media.

### Characterization of loaded SPL microspheres

Nano-microspheres were freeze-dried and glued to a copper sheet with conductive adhesive, sprayed with gold, and then observed with a scanning electron microscope (SEM). 20 mg of L1 or L2 nanospheres were dispersed in 1 ml dichloromethane by ultrasonication. The nanospheres were then freeze-dried and weighed. The drug loading capacity (LC, %) was calculated as the weight of sPL in the microsphere/weight of microsphere × 100% (1). To assess the encapsulation efficiency (EE) of the microspheres, microspheres were precipitated by high-speed ultracentrifugation, and the drug content in the supernatant was measured. EE (%) was calculated as (total drug content—the amount of free drug in the supernatant)/total drug content * 100% (2).

The in vitro release of sPL from the nanospheres was monitored based on the levels of different factors. Briefly, the nanospheres were incubated in PBS at 37 °C, and 100 μl aliquots were withdrawn at pre-determined time points and replaced with the same volume of PBS. The concentrations of the different factors were measured using specific ELISA kits (Jiangsu Jingmei Biotechnology Co. Ltd., Yangcheng, China) according to the manufacturer's instructions. Each measurement was repeated at least 3 times, and the average was calculated.

### Primary chondrocyte isolation and culture

Human tissue samples were collected in accordance with the World Medical Association Helsinki Medical Association Declaration of Ethical Principles Concerning Human Subjects. All participants signed an informed consent form. Chondrocytes were isolated from OA patients undergoing total knee replacement as previously described [15]. Briefly, the cartilage tissue was removed, minced, and digested with type II collagenase for 24 h. The macerated tissue was then filtered through a 200-mesh screen and resuspended in Dulbecco's modified Eagle's medium (DMEM) supplemented with 10% FBS and 1% penicillin/streptomycin. The media was changed every 2–3 days, and the cells were passaged to achieve 80–90% confluency. Only third or fourth-generation cells were used for subsequent experiments.

### Cell proliferation assay

Proliferation rates of the suitably treated cells were measured using the Cell Counting Kit-8 (CCK-8, Dojindo, Japan) as per the manufacturer's instructions. Briefly, the chondrocytes were seeded in 96-well plates at the density of 1 × 10^4^ cells/well, and cultured with 5% (v/v) nanospheres for 1, 3 and 5 days. After washing with phosphate buffer saline (PBS), 10 μl CCK-8 solution was added to each well, and the cells were incubated for 30 min at 37 °C. The absorbance at 450 nm was measured using a microplate reader (Biotek), and each well was measured thrice. The percentage of viable cells was calculated on the basis of the optical density (OD) and expressed relative to that of the untreated controls.

### Live/dead staining

The live/dead cell kit was used to analyze the effect of nanospheres on chondrocyte morphology and viability. The cells were cultured with the nanospheres for 3 days, and then stained with 2 μl ethylenediamine homodimer 1 and 0.5 μl calcein acetylmethoxy in 1 mL DMEM for 30 min [14]. Cells were then washed thrice with PBS and observed under a fluorescence microscope (Leica, Germany).

### Phalloidin staining

The chondrocytes were seeded on a confocal microscope petri dish and cultivate together with the nanospheres for 2 days [14]. Adherent cells were fixed with 4% paraformaldehyde and then permeabilized with 0.2% Triton X-100 for 10 min at room temperature. Subsequently, the cells were stained with FTIR-Phalloidin for 30 min in the dark and then fixed with 4% paraformaldehyde for 20 min at room temperature. Cells were then rinsed with PBS examined using a laser scanning confocal microscope (Leica, Germany).

### Real-time quantitative polymerase chain reaction (RT-qPCR)

Chondrocytes were seeded in 6-well plates at a density of 1 × 10^4^ cells per well and treated with the different nanospheres for 48 h. Total RNA was extracted using the TRIzol reagent (Invitrogen Life Technologies, Carlsbad, CA, USA), and 1 μg per sample was reverse transcribed into cDNA (TaKaRa Bio, Dalian, China). RT-qPCR was conducted on a ABI 7500 sequencing detection system (Applied Biosystems, Foster City, CA, USA). The reaction mix included 20 μl TB Green ® Premix Ex Taq ™ II (RR820A, TaKaRa), 0.8 μl of each primer, and 2 μl diluted cDNA to make up a total volume of 20 µl. The cycling parameters were as follows: 95 °C for 30 s, followed by 40 cycles at 94 °C for 5 s and 60 °C for 30 s. The cycle threshold (Ct) was obtained and normalized to the GPDH level. The 2^−ΔΔCt^ method was used to calculate the relative mRNA level of each target gene. The primers specific for type I collagen, type II collagen, SOX-9, ACAN, and GAPDH are listed in Table 1.

**Table 1:** Primer sequences

Gene	Primers sequence	
	Forward	Reverse
ACAN	CTTCCGCTGGTCAGATGGAC	CGTTTGTAGGTGGTGGCTGT
COL2A1	ATGATTCGCCTCGGGGCTC	CCGGCTTCCACACATCCTTAT
SOX-9	CCCGCTCACAGTACGACTA	TGTAGGTGAAGGTGGAGTAG
GAPDH	TCCAAAATCAAGTGGGGCGA	TGATGACCCTTTTGGCTCCC

### Establishment of animal model

50 SD male rats weighing 180–200 g were obtained from the Department of Laboratory Animal Science, Harbin Medical University. All protocols were approved by the Ethics Committee of the First Affiliated Hospital of Harbin Medical University. The animals were anesthetized with an intraperitoneal injection of 50 mg/kg 3% sodium pentobarbital and 1% lidocaine. OA models were created using the anterior cruciate ligament transection (ACLT) and destabilization of the medial meniscus (DMM) procedures. Briefly, the skin and joint capsule were accessed using the internal patella approach. The anterior cruciate ligament and the medial collateral ligaments were transected, and the internal meniscus was removed to destabilize the knee joint. OA was confirmed by histological examination and imaging 6 weeks after the surgery. Animals were randomly divided into the sham-operated control (joint cavity accessed without removing the medial meniscus and anterior cruciate ligament), normal saline control, and the L0, L1, and L2-treated groups. The rats were treated with intraarticular injections of 2 ml saline or the appropriate microspheres one day after the surgery. Antibiotics were given for 3 consecutive days after the surgery. Tissue samples were collected for the CT examination and histopathological staining at 4 and 12 weeks after treatment.

#### Micro-computed tomography (Micro-CT)

The knee joint was fixed in 4% paraformaldehyde for 24 h and subjected to micro-CT (Micro-CT, QuantumGX, PerkinElmer, USA). The parameters were as follows: time 14 min, tube voltage 90 kV, and tube current 88uA. Sequential 2D images were reconstructed from the 3D images using the Analyze 11.0 software (PerkinElmer, Waltham, MA, USA).

#### Histology

The fixed tissues were stained with HE and Safranin O-Fast Green according standard protocols. Briefly, the specimens were decalcified in 10% Ethylene Diamine Tetraacetic Acid (EDTA) for two weeks, embedded in paraffin, cut into 4 μm thick sections, and stained with HE and Safranin-O/Fast Green staining dyes (Solarbio, Beijing, China). Cartilage degeneration was evaluated by the Histopathology Assessment System of the International Osteoarthritis Research Society (OARSI) [16]. The samples were scored by three independent observers blinded to the grouping. The average OA scores were calculated and used as the representative scores of the knee joint [17]. The in-situ expression of proteoglycan and type I collagen was analyzed by immunohistochemistry using standard protocols. The stained sections were observed under a fluorescence microscope (Leica, Germany). Image J software was used to measure the cartilage thickness of each joint at 20 different points in the damaged area, and the average was calculated.

#### Statistical analysis

The data is presented as mean ± standard deviation (SD). One-way analysis or two-way analysis of variance (ANOVA) were used to compare between multiple groups by Tukey's. All experiments were repeated thrice. *P* < 0.05 was considered statistically significant.

## Results and discussion

### Morphological characterization

**Fig. 1:**
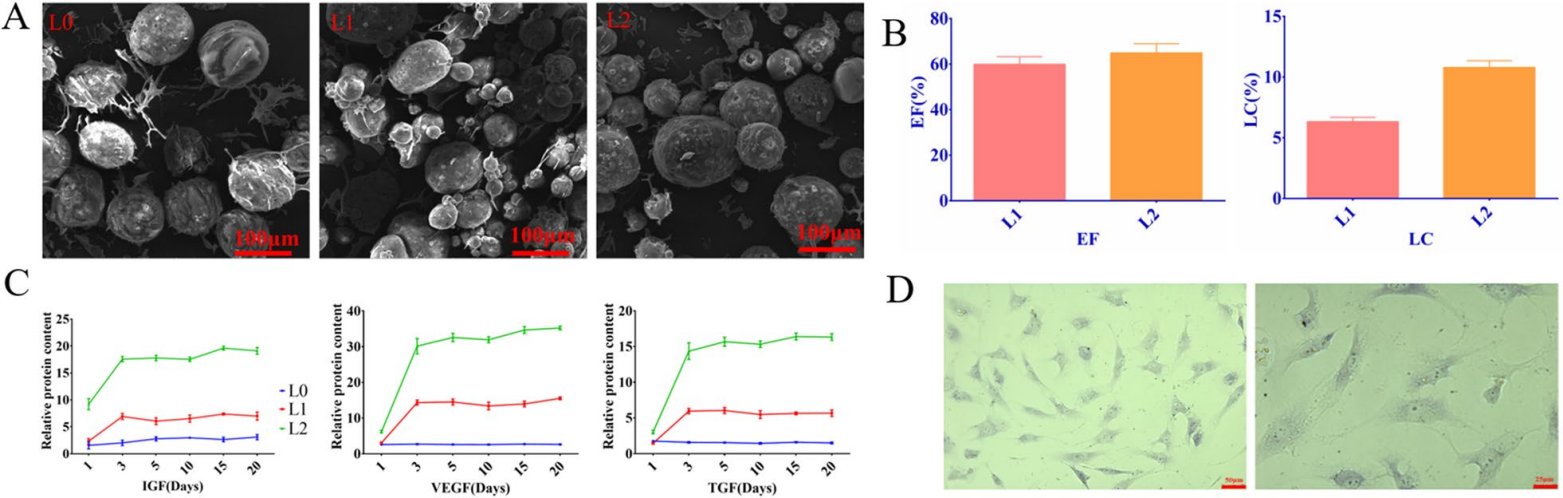
Characterization of loaded sPL microspheres. **a** An SEM image of the indicated microspheres. **b** Load rate and encapsulation rate of L1 and L2 microspheres. **c** The time-dependent release of IGF, TGF and VEGF from L0, L1 and L2. **d** Light micrograph of chondrocytes

As shown in the SEM images in Fig. 1a, nanospheres prepared using the liquid phase separation method were round and uniform. The slightly larger size of the L2 nanospheres can be attributed to differences in the content of ionic co-monomers, which are known to affect the size, shape and uniformity of nanospheres. For instance, an increase in the number of comonomers reduces nanosphere size and increases their surface stability [18]. Furthermore, increasing the reaction time or stirring frequency may also increase or decrease the size of nanospheres.

### sPL-loaded nanospheres steadily release growth factors in vitro

Hydrogel nanospheres are effective drug nanocarriers owing to their hydrophilicity and network structure. Thus, sPL released from chitosan, gelatin and PLGA nanospheres can promote autophagy of damaged chondrocytes, increase secretion of anti-inflammatory markers and protect healthy chondrocytes from apoptosis [15]. The LC and EE values of L1 nanospheres were 6.32% and 59.94% respectively, and that of L2 were 10.79% and 64.96% (Fig. 1b), respectively, indicating that the nanospheres synthesized in this study effectively encapsulated sPL.

As shown in the drug release curve in Fig. 1c, growth factors were rapidly released from the sPL-loaded nanospheres within the first 72 h, and stabilized thereafter. At 37 °C, the amount of growth factors released from the L2 microspheres was twofold higher than that of the L1 nanospheres. Growth factor levels were consistently higher in the L2 group over a period of 10 days. As expected, no growth factors were released from the L0 particles. Thus, sPL-loaded nanospheres released the cargo in a controlled manner and can act as a means of replenishing synthetic synovial lubricant in OA. This is in line with a previous study showing the sustained release of biological factors from biopolymers [19].

### Microsphere cytotoxicity and its effect on chondrocytes

**Fig. 2:**
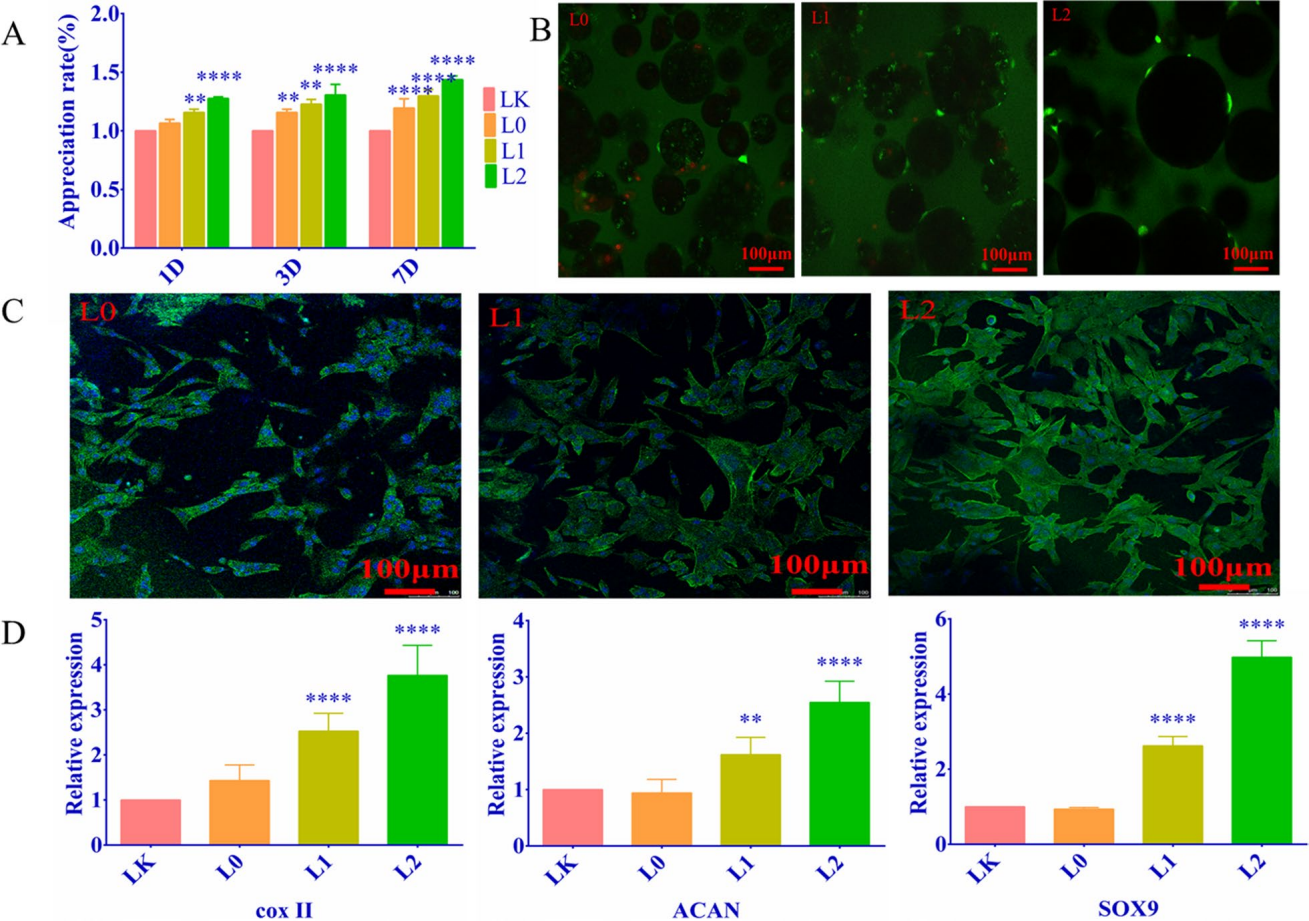
**a** Chondrocyte cell proliferation when cultured with microspheres for 1, 3, and 7 days. Compared with the blank group, the L0 group promoted cell proliferation on the 7th day and the L1 group significantly promoted cell proliferation. Hydrogel loaded with sPL significantly promoted chondrocyte proliferation. **b** The proportion of viable cells following treatment with different microspheres (Green represents living cells, red represents dead cells); **c** Cell changes in cells cultured with microspheres as shown using ring pen ghost tire staining (Green indicates cell cytoskeleton, while blue indicates the nucleus); **d** The relative expression of chondrogenesis genes in the indicated groups. Compared with the blank group, the expression levels of type II collagen, ACAN, and SOX9 in L1 and L2 groups were significantly increased. (LK: no material and no sPL) **P < 0.0.1; ***P < 0.001; ****P < 0.0001

To examine the clinical impact of the synthesized nanospheres, we evaluated their effects on cultured primary chondrocytes. The extracted chondrocytes appeared purplish-red after toluidine blue staining, indicating the presence of proteoglycans and glycosaminoglycans (Fig. 1d). As shown in Fig. 2a, the L0 nanospheres slightly increased the proportion of viable cells and the proliferation rates after 1 day of culture. In addition, all nanospheres significantly increased the number of chondrocytes on the 3rd and 7th days. Based on these findings, we conclude that the microspheres were biocompatible, promoted chondrocyte proliferation, and were not toxic.

Furthermore, live/dead cell staining showed that most chondrocytes were viable after 3 days of culture with the nanospheres (Fig. 2b). Live cells are stained green, and the dead cells appear red. The cellular density increased depending on the sPL concentration. In addition, the viability of the microsphere-treated chondrocytes was higher than that of untreated controls, indicating that microspheres had nodular effects on chondrocytes. However, the number of viable cells did not change in a time-dependent manner in any of the groups. As shown in Fig. 2c, Phalloidin staining further indicated the chondrocytes exposed to sPL demonstrated enhanced viability and preserved morphology.

The pathogenesis of OA involves multiple cellular, biochemical, and molecular changes. For instance, affected chondrocytes typically overexpress catabolic genes and show low levels of anabolic genes. Osteoarthritic progression is associated with downregulation of the chondrogenic transcription factor SOX9 and cartilage-specific matrix molecule type II collagen (COL2A1), along with upregulation of ACAN and type I collagens (COL1A1 and COL1A2) in the chondrocytes, indicating a shift to the fibrocartilage (fibroblast) phenotype[20, 21]. As shown in Fig. 2d, SOX-9, ACAN and COL2A2 mRNAs were significantly upregulated I the L1 and L2 groups compared to that in the L0 group, and in the L2 versus L1 group (*P* < 0.001). All results indicated that sPL-loaded nanospheres have significant chondroprotective abilities, which may restore cartilage function during OA.

### sPL-encapsulated hydrogel nanospheres prevented cartilage defects in the OA model

**Fig. 3:**
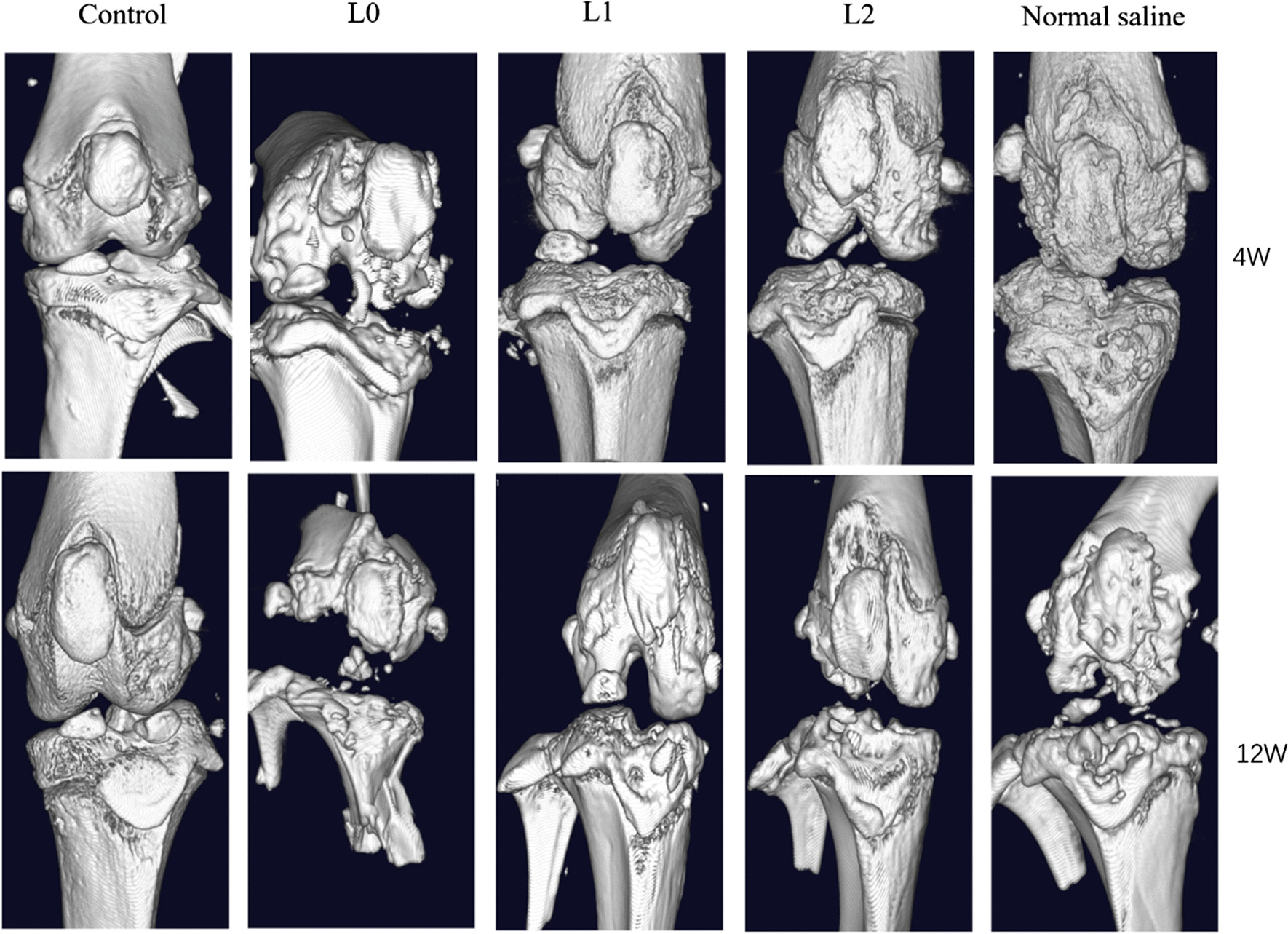
Micro-CT scans showing the effect of microspheres on bone and articular cartilage injury. (Control group: Joint cavity was surgically accessed without any procedure done; L0: L0 microspheres injected after the ACLT and DMM procedures; L1, L1 microspheres injected after the ACLT and DMM procedures; L2, L2 microspheres injected after the ACLT and DMM procedures; Normal saline group: normal saline microspheres injected after the ACLT and DMM procedures)

**Fig. 4:**
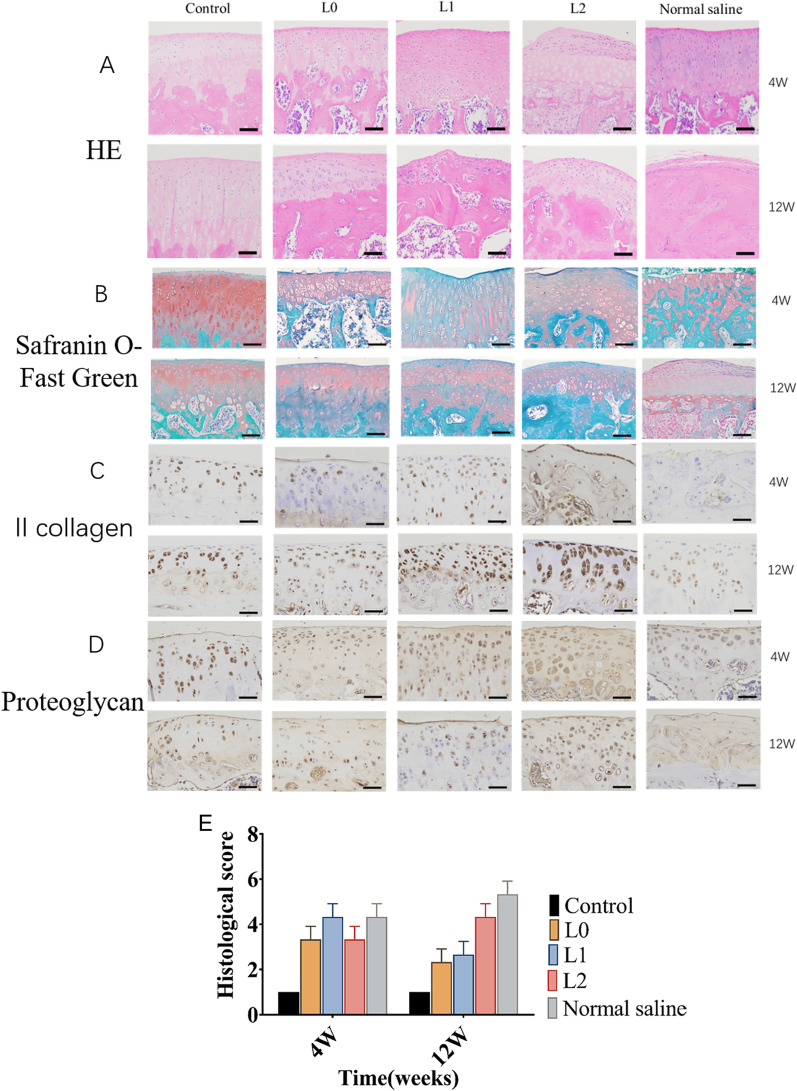
Histological and biochemical analyses were used to evaluate the protective and restorative effects of microsphere treatment on the cartilage of OA rats. Typical images of knee joints. Histological staining was performed at 4 and 12 weeks after surgery. **a** HE staining demonstrates a clear boundary between the cartilage and subchondral layer; **b** Safranin-O Fast Green staining demonstrates cartilage matrix and chondrocyte plasma (stained red), and muscle, collagen fibers, and bone tissue (stained gray-green); **c** II collagen staining demonstrates gray-black small particles which represent type II collagen fibers; **d** Chondrocyte proteoglycan staining demonstrates gray-black small particles which represent proteoglycans; **e** OARSI histological scoring of the cartilage at 4 and 12 weeks after surgery. (Control group: Joint cavity was surgically accessed without any procedure done; L0: L0 microspheres injected after the ACLT and DMM procedures; L1, L1 microspheres injected after the ACLT and DMM procedures; L2, L2 microspheres injected after the ACLT and DMM procedures; Normal saline group: normal saline microspheres injected after the ACLT and DMM procedures)

The ability of the sPL-encapsulated nanospheres to promote cartilage repair was evaluated in a rat OA model (Fig. 3). Micro-CT showed that the ACLT and DMM procedures successfully induced OA within 6 weeks. L0, L1 and L2 nanospheres or saline were respectively injected into the knee joints. Ten weeks after surgery, the joint surface of the sham-operated animals was completely smooth. In contrast, saline-treated osteoarthritic rats showed the erosion and denudation associated with OA, including loss of bone surface matrix and hardened bone. L0 nanospheres partially reduced the degenerative changes, whereas the joint surfaces of the L1 and L2 groups were largely smooth. After 18 weeks post-operation, the saline-treated animals showed extensive cartilage destruction with matrix loss and surface exfoliation. In addition, the joint surface in the L0 groups were not smooth, and showed reduced joint space and multiple osteophytes. In contrast, L1 and L2 nanospheres significantly smoothened the joint surface, with a visible joint space and only a few osteophytes (Fig. 4a). Furthermore, the L2 nanospheres significantly reduced cartilage loss and surface layer delamination, and increased protein glycation and filling up of the deeper cartilage layers. Safranin O-Fast Green staining (Fig. 4b) further showed significant cartilage repair in the L2-treated group. The histological score (2 ± 0.81) of the L2 group was also lower than that of the normal saline group (4.33 ± 0.47), and similar to that of the sham-operated controls (Fig. 4e). In addition, the average thickness of the hyaline cartilage in the damaged area was higher in L2 versus the sham-operated group 10 weeks after surgery, whereas no significant difference was observed between the L1 and L0 groups. The concentration and spatial distribution of type II collagen differs across the depth of cartilage tissue, and accounts for 90%-95% of the collagen network [22]. The aggrecan II collagen levels were significantly reduced in the cartilage matrix of saline-treated rats, and showed only a slight reduction in the animals treated with L2 nanospheres (Fig. 4c). Furthermore, chondrocyte proteoglycan staining showed similar results (Fig. 4d).

These results show that L0 nanospheres can effectively protect osteoarthritic cartilage, and their regenerative effects are augmented with the inclusion of sPL. CS is known to enhance cartilage formation by increasing the density of articular cartilage chondrocytes [23]. Furthermore, the presence of GAG further enhances the cartilage forming ability of chitosan hydrogels [24]. FGF also stimulates the proliferation of chondrocytes, and induces the expression of type II collagen and matrix proteins [25]. Gigout et al. found that FGF increases the number of matrix-producing chondrocytes in 3D culture, improves the proportion of type II collagen, and stimulates the production of a transparent extracellular matrix [26]. Therefore, in addition to the sPL-related bioactive factors released from the nanospheres, hydrolyzed CS from the L1 and L2 nanospheres may also contribute to chondroprotection.

To summarize, a combination of biological factors and matrix-simulating hydrogel nanospheres can promote cartilage repair in OA animal models. The initial rapid release of bioactive factors acts to reduce pain, whereas a sustained release results in cartilage repair. We conclude that intraarticular injection of nanospheres represents a promising treatment for OA.

## Conclusion

Nanospheres were successfully prepared by liquid phase separation of PLGA and CS/gelatin. The initial burst and later sustained release of biological factors from the encapsulated sPL enhanced chondrocyte proliferation and restored osteoarthritic cartilage to healthy cartilage. sPL-loaded nanospheres are promising candidates that may reverse cartilage degeneration seen in OA.

## Abbreviations

Rt-PCR: Reverse transcription-polymerase chain reaction
ACAN: Aggrecan
PGLA: Poly (DL-lactide-glycolide-glycolide acid)
CS: Chitosan
GAG: Glycosaminoglycan
TGF: Transforming growth factor
OA: Osteoarthritis
IGF-1: Insulin-like growth factor
EE: Encapsulation efficiency
LC: Loading capacity
DMEM: Dulbecco's modified Eagle’s medium
CCK-8: Cell counting kit-8
SEM: Scanning electron microscope
Micro-CT: Micro-computed tomography
EDTA: Ethylene diamine tetraacetic acid
